# Genetic testing in Indian patients with Prader-Willi syndrome using methylation specific multiplex ligation dependent probe amplification (MS-MLPA)

**DOI:** 10.1186/1687-9856-2015-S1-P75

**Published:** 2015-04-28

**Authors:** Vaman Khadilkar, Varsha Jagtap, John Mathew, Nikhil Phadke, Kavita Khatod, Ketki Kelkar-Ramanan, Anuradha Khadilkar

**Affiliations:** 1 Hirabai Cowasji Jehangir Medical Research Institute, Jehangir Hospital, Pune, Maharashtra, India; 2 KEM Hospital, Pune, Maharashtra, India; 3 Providence Endocrine & Diabetes Speciality Centre, Trivandrum, Kerala, India; 4 GenePath Dx, Pune, Maharashtra, India

## 

Prader-Willi syndrome (PWS) is caused by loss of function of genes on chromosome 15; most cases occur when a segment of the paternal chromosome 15 is absent/inactivated. Recently few cases have been identified with truncating mutations in MAGEL2 gene (Chromosome 15). We present clinical features and molecular genetic analysis on 6 patients with features of PWS using MS-MLPA.

Four boys and 2 girls (age 8-15 years) presenting with obesity, facial features of PWS, developmental delay, snoring and hypogonadism were referred for genetic testing. DNA was extracted from peripheral blood and MS-MLPA was carried out using MS-MLPA Probemix (ME028) from MRC Holland[1]. Data analyses were performed using Coffalyser Software (MRC Holland). MS-MLPA was used to detect copy number changes and determine CpG island methylation status of 15q11.3-13 locus in semi-quantitative manner.

Of six patients, no genetic defect was detected in one. Two patients’ DNA on MLPA analysis showed 50% reduction in peak intensities (compared to healthy control) of 29 probes in the targeted 15q11.3-13 region indicating heterozygous deletion of that region. The single copy that was present in these patients was imprinted and likely to be silenced. In the fourth patient, no detectable deletion was found, however, comparative MS-MLPA analysis of this sample pre- and post- methylation-specific restriction digestion showed approximately 100% relative methylation for the 5 probes specific to the imprinted regions (these are 50% in healthy individuals). Both copies of the analyzed region were imprinted and likely to be silenced (most likely due to maternal uniparental disomy). For the last two patients, while no deletion was found, both samples showed hypomethylation (0% methylation) of one probe in necdin-encoding gene (NDN) locus (for this locus, expected methylation for healthy controls is between 30%-50%). We are investigating the relevance of these data.

Our MS-MLPA results in 6 patients with clinical features of PWS indicate that two cases had deletions, one had a maternal uniparental disomy or an imprinting defect, two had hypomethylation of the NDN locus and one did not show any defect.
